# The Therapeutic Potential and Molecular Mechanism of Isoflavone Extract against Psoriasis

**DOI:** 10.1038/s41598-018-24726-z

**Published:** 2018-04-20

**Authors:** Hsin-Ju Li, Nan-Lin Wu, Gon-Ann Lee, Chi-Feng Hung

**Affiliations:** 1 Department of Chemistry, Fu Jen Catholic University, New Taipei City, 24205 Taiwan; 2 School of Medicine, Fu Jen Catholic University, New Taipei City, 24205 Taiwan; 3 Graduate Institute of Biomedical and Pharmaceutical Science, Fu-Jen Catholic University, New Taipei City, 24205 Taiwan; 4 Department of Fragrance and Cosmetic Science, Kaohsiung Medical University, Kaohsiung, 80708 Taiwan; 5 Department of Medicine, Mackay Medical College, New Taipei City, 25245 Taiwan; 6 Department of Dermatology, Mackay Memorial Hospital, Taipei, 10449 Taiwan; 7 Mackay Junior College of Medicine, Nursing, and Management, New Taipei City, 25245 Taiwan

## Abstract

Psoriasis is a common inflammatory disease. It affects 1–3% of the population worldwide and is associated with increasing medical costs every year. Typical psoriatic skin lesions are reddish, thick, scaly plaques that can occur on multiple skin sites all over the body. Topical application of imiquimod (IMQ), a toll-like receptor (TLR)7 agonist and potent immune system activator, can induce and exacerbate psoriasis. Previous studies have demonstrated that isoflavone extract has an antioxidant effect which may help decrease inflammation and inflammatory pain. Through *in vivo* studies in mice, we found that the topical application to the shaved back and right ear of mice of isoflavone extract prior to IMQ treatment significantly decreased trans-epidermal water loss (TEWL), erythema, blood flow speed, and ear thickness, while it increased surface skin hydration, and attenuated epidermal hyperplasia and inflammatory cell infiltration. Through *in vitro* experiments, we found that isoflavone extract can reduce IL-22, IL-17A, and TNF-α-induced MAPK, NF-κB, and JAK-STAT activation in normal human epidermal keratinocytes. At the mRNA level, we determined that isoflavone extract attenuated the increased response of the TNF-α-, IL-17A-, and IL-22- related pathways. These results indicate that isoflavone extract has great potential as an anti-psoriatic agent and in the treatment of other inflammatory skin diseases.

## Introduction

Psoriasis is a common chronic immune-mediated inflammatory disease with a prevalence of 1–3% worldwide^1–3^, and is characterized by red, scaly, raised plaques^4,5^. The pathologic features of psoriatic skin lesions are due to hyperproliferation of epidermal keratinocytes, aberrant differentiation of epidermal keratinocytes, infiltration of distinct types of inflammatory and immune cells, and over-activation of angiogenesis^5^. Genetic and immunological studies have noted the critical role of cytokines in the pathogenesis of psoriasis^6,7^. Psoriatic inflammation was initially thought to be mediated by T-helper 1 (TH1) cells; however, more recent studies have revealed that TH17 cells play an indispensable role in the progression of the disease by producing IL-17 or IL-22^5^. The involvement of these cytokines in psoriasis has also been revealed through the therapeutic silencing of key immune system components, such as TNF-α, IL-17, IFN-γ, IL-22, and the Th17/IL-23 axis. This approach has been highly successful and has demonstrated the importance of cytokine networking in psoriasis. Further, it strongly supports the proposed immunopathogenic mechanisms of psoriasis^6–11^.

**Figure 1:**
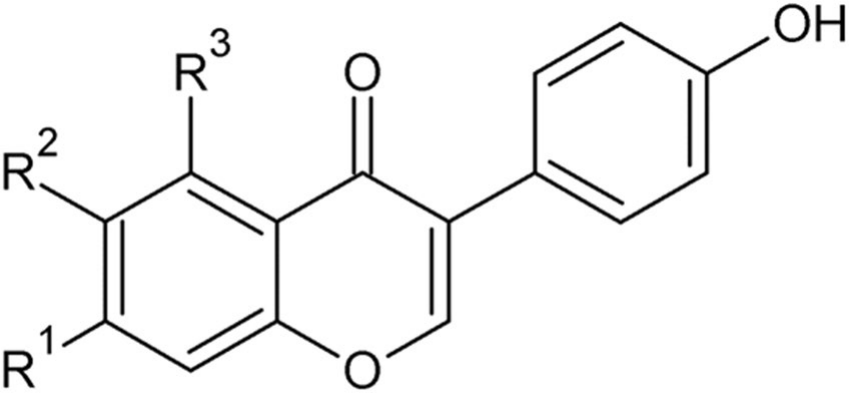
The structure of isoflavone.

Soybean is a common agricultural crop often used in Asian cuisine. It contains many nutrients including plant protein, saponins, phytosterol, oligosaccharides, and isoflavones^12,13^, making it beneficial to the human body. There are 12 different soybean isoflavone isomers. Genistein and daidzein are both major isoflavones but genistein is the most widely investigated^14,15^. Isoflavones (Fig. 1) are a type of phytoestrogen, and have similar molecular structures to animal oestrogen. This leads to weak oestrogenic effects^15^ including the treatment of menopausal symptoms^16^ and prostate cancer^17^, bone strengthening^18^, and reduction of breast cancer risk^19^. Some immune cells, in turn, such as thymocytes, lymphocytes, and macrophages have been reported to contain oestrogen receptors. These receptors bind oestrogen that can inhibit over-activation of immune cells along with delayed hypersensitivity reactions^20^. Isoflavones have been reported to bind to oestrogen receptors and to increase eicosanoid function, and play effectiveness of oestrogen. They also possess antioxidant properties and can scavenge reactive oxygen species, leading to a strong anti-inflammatory effect^21–23^. In this study, we applied the rodent model of psoriasis to evaluate the *in vivo* protective effects of isoflavone extract in alleviating imiquimod-induced inflammatory effects on the development and physiological parameters of mice. Further, we evaluated the efficacy of isoflavone extract in preventing epidermal keratinocytes from psoriatic activity.

## Results

### Isoflavone extract attenuates skin inflammation and hyperplasia in a murine IMQ–induced model

**Figure 2:**
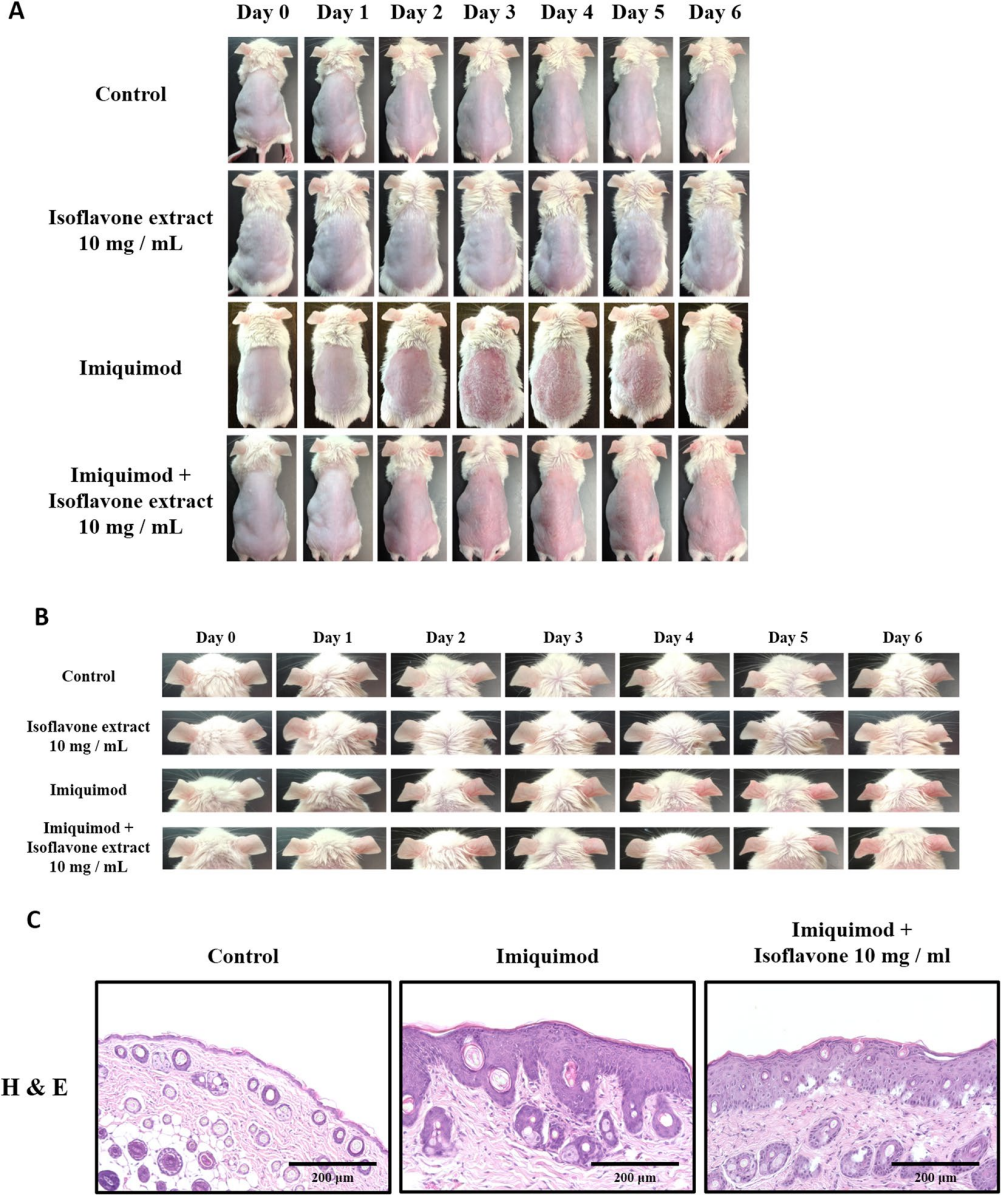
Isoflavone extract ameliorates imiquimod (IMQ)-induced skin inflammation. Isoflavone extract was topically applied 1 hour before IMQ or vehicle cream administration for 6 consecutive days. (**A**) Phenotypical presentation of mouse skin after 6 days of treatment. (**B**) Macroscopic appearance of the ear during 6 days of the experiment. (**C**) Histological analyses of mouse skin are after haematoxylin and eosin (H&E) staining of skin biopsies. In each group, mice were applied with vaseline or IMQ after pretreatment with isoflavone extract.

We studied the potential beneficial effect of isoflavone extract in a murine IMQ-induced psoriasis-like skin inflammation model in which IMQ, a TLR7/8 agonist, was applied to the whole back of the mice daily over 6 days. This repetitive application of IMQ onto mouse skin led to psoriasis-like inflammation with significant thickening, redness, and scaling caused by keratinocyte hyperproliferation and leukocyte infiltration into the skin (Fig. 2A,B).

Histological analysis of lesion skin biopsies using H&E staining showed a decrease of inflammatory cell infiltrate in isoflavone extract-pretreated mice compared with IMQ-treated mice. In addition, isoflavone extract treatment prevented the characteristic epidermal hyperplasia induced by IMQ (Fig. 2C).

**Figure 3:**
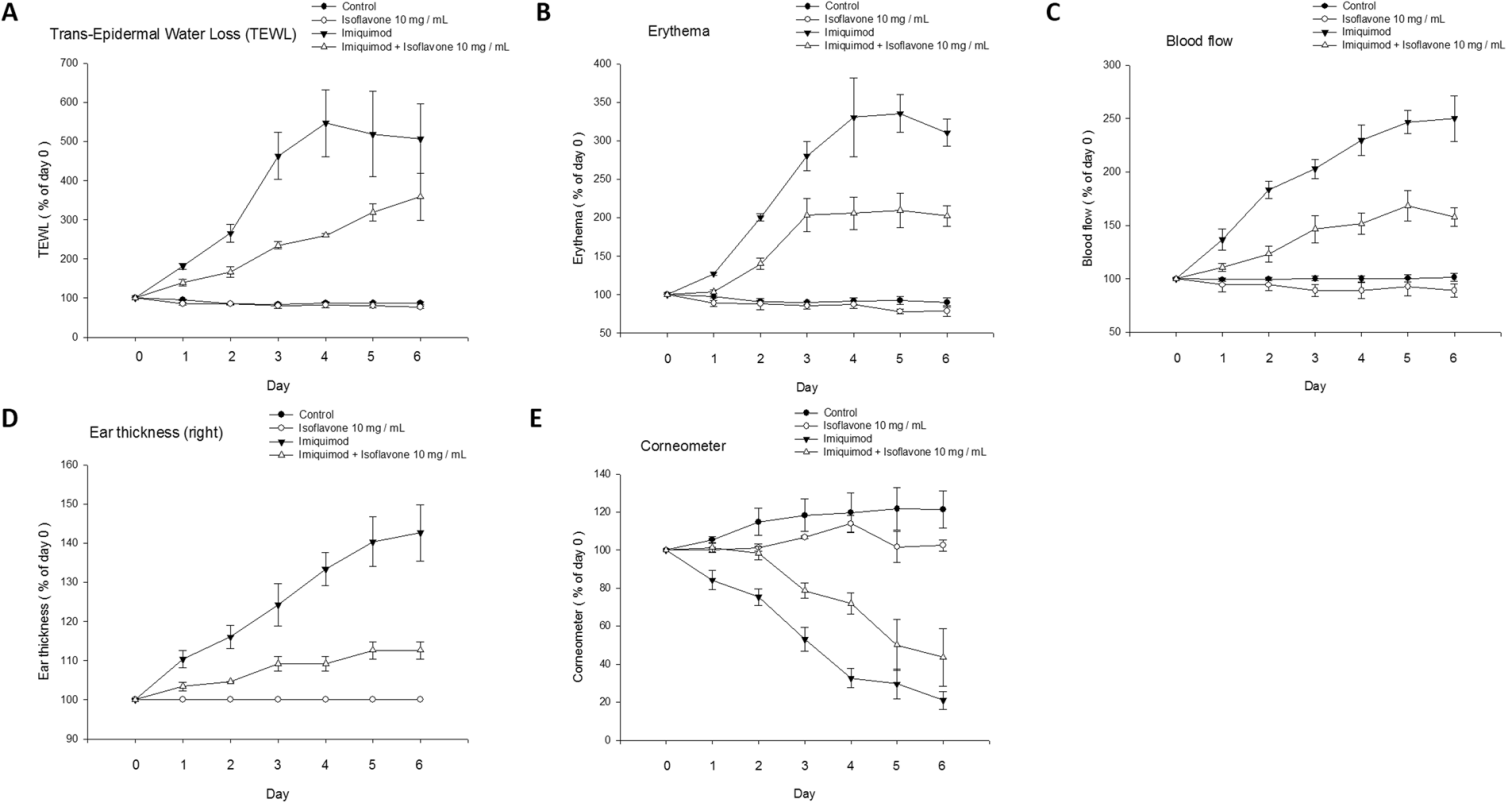
Change in physiological parameters on mice skin surface after treatment with isoflavone extract. Analysis of the change in ear thickness, trans-epidermal water loss (TEWL), erythema, and blood flow values in different treatments. A gradual decrease in the value of the parameters was observed after pre-treatment with isoflavone extract before stimulation with IMQ (**A**–**D**). Corneometer a tool used to determine the hydration level of the skin surface and the value of the corneometer increases with isoflavone extract treatment after IMQ stimulation (**E**). (**A**) TEWL (**B**) erythema (**C**) blood flow (**D**) ear thickness (**E**) corneometer measurement. Data are the mean ± SEM from at least six independent experiments.

Topical pre-treatment with isoflavone extract prior to IMQ application ameliorated lesion formation in a dose-dependent manner and significantly reduced the development of erythema at the site of application (Fig. 3B). There were no significant changes after treatment with isoflavone extract alone. Moreover, our data showed that the treatment with isoflavone extract inhibited the TEWL, erythema, blood flow, ear thickness, and increased a corneometer in a dose-dependent manner. There were no significant differences in melanin or pH value between the IMQ treatment alone and mice treated with the isoflavone extract before IMQ treatment (Fig. 3A–E).

### Isoflavone extract is not cytotoxic to NHEKs

**Figure 4:**
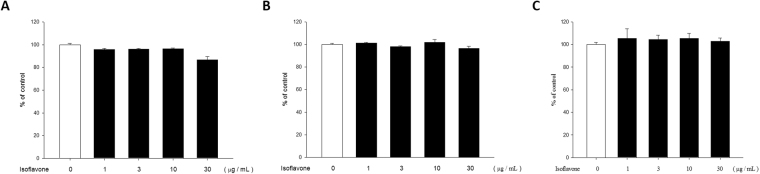
Normal human epidermal keratinocyte cells show viability under different concentrations of isoflavone extract treatment. (**A**) Cell viability in normal human keratinocyte cells was determined via MTT assay after incubation with 1, 3, 10, and 30 μg/mL isoflavone extract, respectively, for 24 h. (**B**) Cell viability in NHEKs was determined via crystal violet assay and (**C**) trypan blue assay. Values represent means of optical density values measured at 550 nm ± standard error of mean (SEM) from at least three independent experiments.

The cytotoxic effect of isoflavone extract on NHEKs was determined via MTT, trypan blue, and crystal violet assays. Treatment with isoflavone extract in a concentration range of 1–10 μg·mL^−1^ for 24 hours did not reduce cell viability (Fig. 4). However, the trial of 30 μg·mL^−1^ isoflavone extract showed slight cytotoxicity. We, therefore, used isoflavone extract at concentrations of 1–10 μg·mL^−1^ in our experiments.

### Isoflavone extract inhibits TNF-α-, IL-17A-, and IL-22-induced MAPK pathway phosphorylation in NHEKs

**Figure 5:**
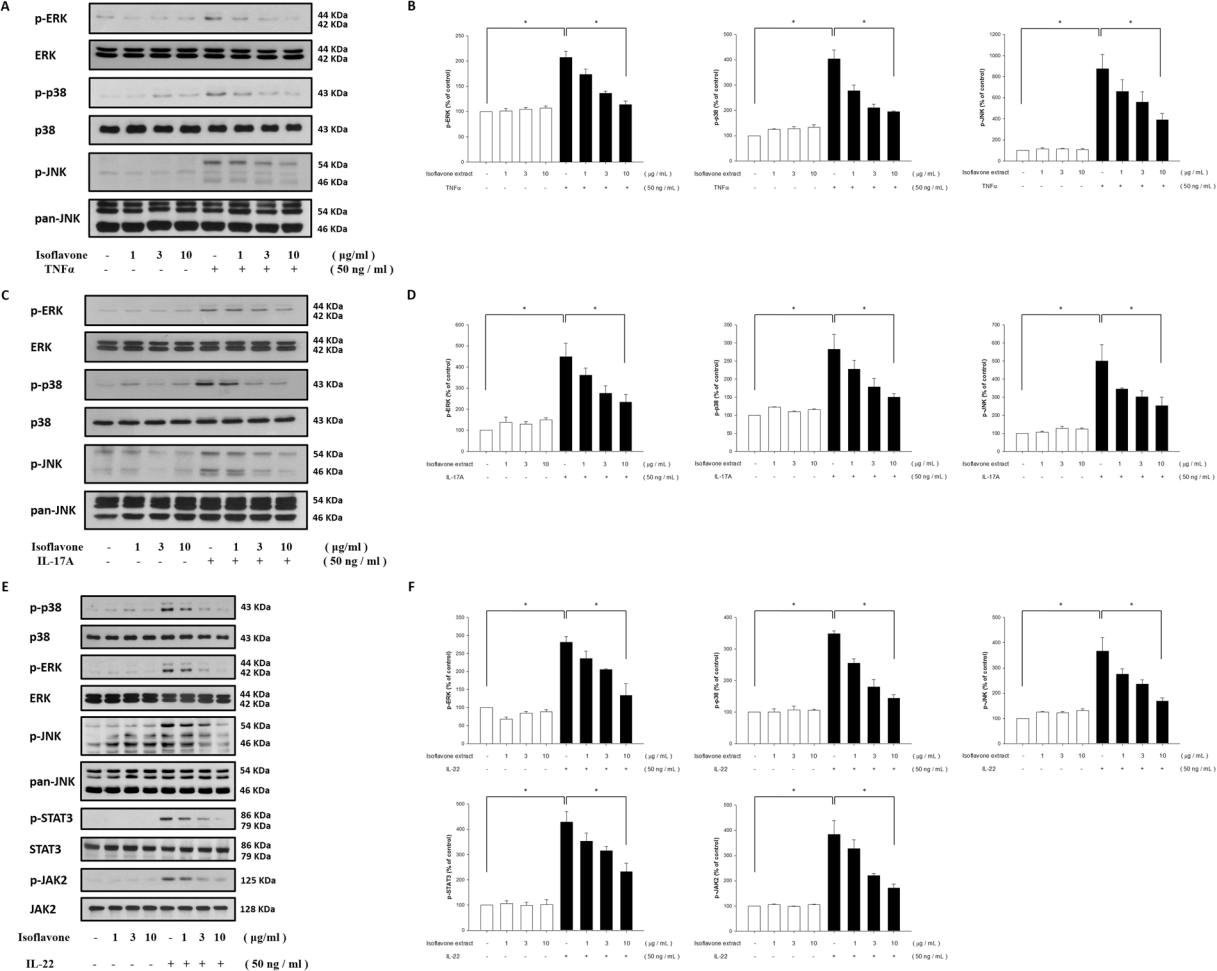
Isoflavone extract inhibits TNF-α-, IL-17A-, and IL-22-induced MAPK and JAK/STAT pathway activation in human primary keratinocytes. Primary keratinocytes were pretreated with different doses of isoflavone extract for 24 hours and then treated with TNF-α, IL-17A, or IL-22. After treatment of TNF-α (**A**), IL-17A (**C**), or IL-22 (**E**), with the quantification data shown in the right panel (**B**,**D**,**F**), the total protein was extracted from the cells and associated protein expression were determined via western blotting. Data are the mean ± SEM from at least three independent experiments. *P < 0.05, indicating significant effects of isoflavone extract as compared to control.

MAP kinase signalling is critically involved in TNF-α-activated pathways and regulates various downstream effects in skin cells. This study, therefore, investigated possible signalling pathways modulated by isoflavone extract. A western blot analysis of NHEKs performed after they were pre-incubated with isoflavone extract for 24 h and stimulated with TNF-α (50 ng·ml^−1^) showed that the changes in phosphorylated ERK, p38, and c-Jun N-terminal kinase (JNK) expression were induced by TNF-α alone. Isoflavone extract-pretreatment suppressed the production of TNF-α-induced ERK, p38, and JNK phosphorylation in a dose-dependent manner (Fig. 5A).

To assess the activation of MAPK following exposure to IL-17A (50·ng mL^−1^), we examined phosphorylation levels of p38, ERK, and JNK as the relevant downstream molecules via western blot. Our results showed a significant increase in the phosphorylation of p38, ERK, and JNK after treatment with IL-17A alone. These increases could be significantly lowered with isoflavone extract in a dose-dependent manner (Fig. 5B). Our results demonstrate that isoflavone extract can effectively reduce the activation of MAPK.

The JAK-STAT pathway is a classical signal transduction pathway for numerous cytokines and growth factors^24^. IL-22 activates the JAK/STAT, ERK, JNK, and p38 MAPK pathways^25^. Therefore, we assessed how IL-22-induced activation is affected by the presence of isoflavone extract in NHEKs using western blot analysis. The analysis of IL-22-induced molecular cascades demonstrated that JAK/STAT and MAPK pathways were rapidly induced in NHEKs and that the phosphorylations of STAT3, JAK2, ERK, JNK, and p38 were inhibited by treatment with isoflavone (Fig. 5C).

### Isoflavone extract inhibits TNF-α- and IL-17A-induced NF-κB pathway activation in NHEK

**Figure 6:**
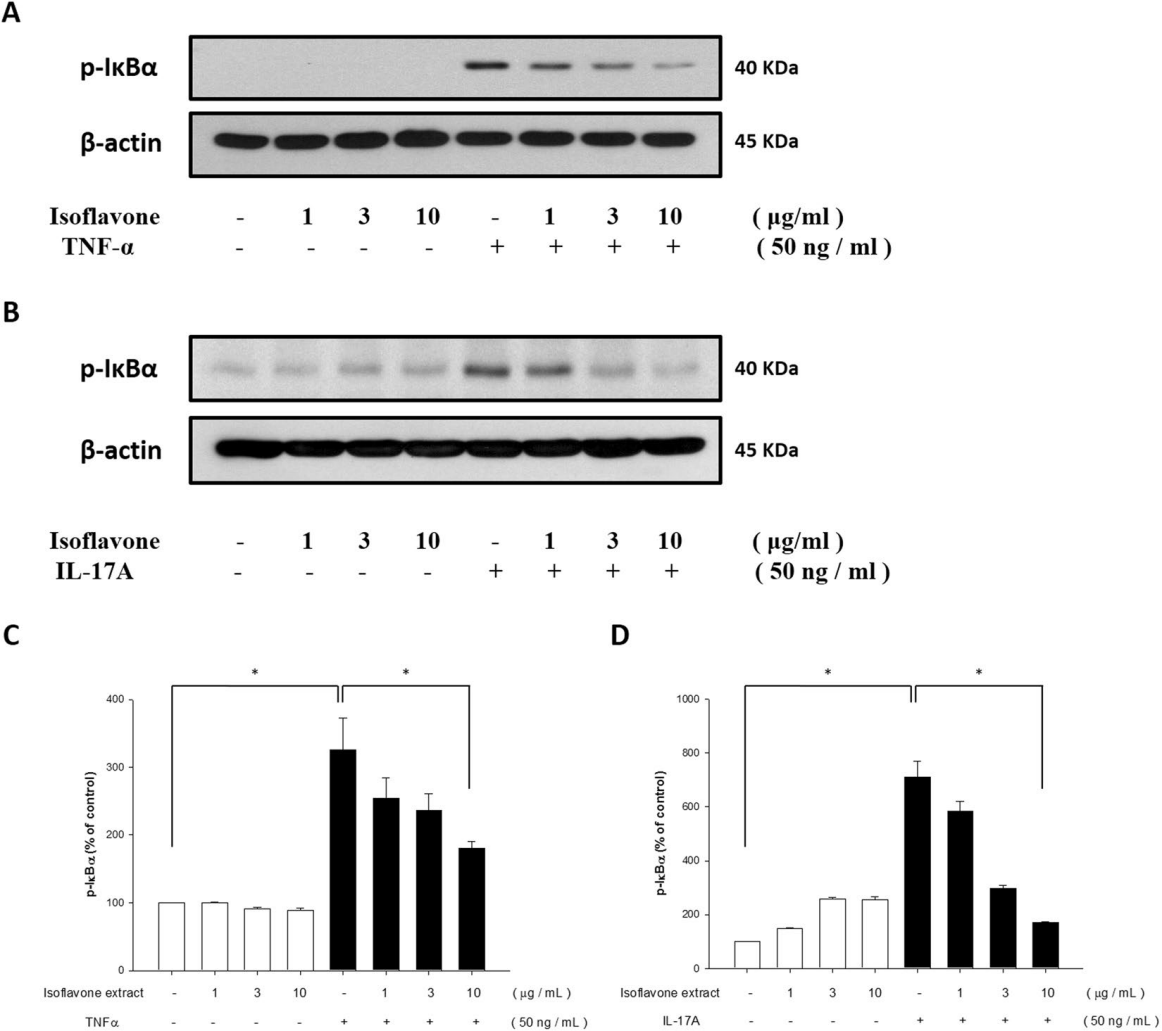
Isoflavone extract inhibited TNF-α- and IL-17A-induced NF-κB phosphorylation in human primary keratinocytes. Primary keratinocytes were pretreated with different doses of isoflavone extract for 24 hours and then treated with TNF-α or IL-17A. After treatment with TNFα (**A**) or IL-17A (**C**), with the quantification data shown in the right panel (**B**,**D**), the total protein was extracted from the cells and IκBα expression was determined via western blotting. Data were the mean ± SEM from at least three independent experiments. *P < 0.05, indicating significant effects of isoflavone extract as compared to control.

Since phosphorylation and degradation of the IκB protein, especially the α isoform (IκBα) are critical steps in the activation of the NF-κB signalling pathway^26^, we measured the effect of isoflavone extract on the TNFα- and IL-17A-induced phosphorylation and degradation of IκBα. Once NHEKs are stimulated by TNF-α or IL-17A, IκBαs are phosphorylated and degraded rapidly. As shown in Fig. 6, treatment of NHEKs with TNF-α or IL-17A increased the phosphorylation and degradation of IκBα, and this stimulation was prevented significantly by pre-treatment with isoflavone extract (Fig. 6). These results indicate that isoflavone extract influences NF-κB activation by regulating the phosphorylation and degradation of IκBα.

### Isoflavone extract inhibits TNF-α-, IL-17A-, and IL-22-induced mRNA expression in NHEKs

**Figure 7:**
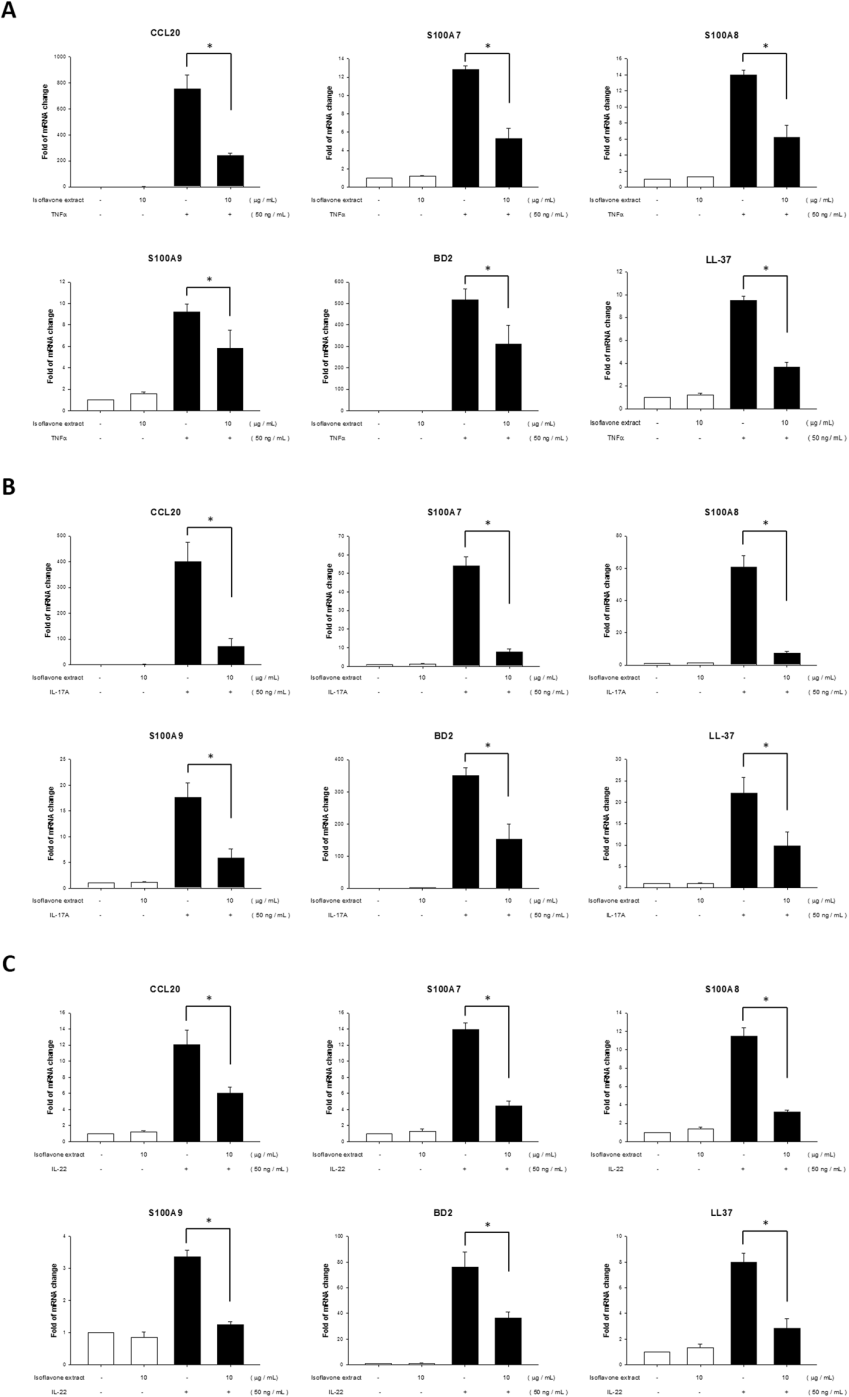
Real-time polymerase chain reaction (PCR) quantification of fold changes in CCL20, S100A7, S100A8, S100A9, hBD2, and LL-37 transcripts in human primary keratinocytes after the addition of TNFα, IL-17A, and IL-22 recombination proteins. Primary keratinocytes were pretreated with different doses of isoflavone extract for 24 hours and then incubated with TNFα (**A**), IL-17A (**B**), and IL-22 (**C**) for 6 or 8 h. Total RNA was then isolated and mRNA analysis performed via real-time RT-PCR. Data are expressed as fold induction of relevant mRNAs sequences as compared to untreated controls. Data are mean ± SEM from at least three independent experiments performed in triplicate; *P < 0.05 determined by Student’s t-test.

Antimicrobial peptides and proteins (AMPs) are a diverse group of small molecules (12–50 amino acids), which are ubiquitously distributed in mammals, insects, frogs, and which can even be found in plants and invertebrates^27^. The main function of AMPs is to resist and kill pathogenic microorganisms such as bacteria, fungi, and viruses. In general, AMPs are concentrated in animal body surfaces, such as the skin and gastrointestinal tract^28,29^. In patients with psoriatic lesions, there are three subclasses of AMPs, including cathelicidin, S100 proteins, and defensins, which are highly expressed, and which are considered to play an important role in the pathogenesis of psoriasis^30,31^. RT-qPCR analysis of NHEKs revealed that during the development of psoriatic inflammation induced by TNF-α, IL-17A, or IL-22, increased levels of the AMPs CCL20, S100A7, S100A8, S100A9, hBD2, and LL-37 modulated and triggered host immune response. Moreover, we found that expression of CCL20, S100A7, S100A8, S100A9, hBD2, and LL-37 significant decreased after pretreatment with isoflavone extract, compared with the group treated with TNF-α, IL-17A, or IL-22 alone (Fig. 7). These data indicate that isoflavone extract attenuated the TNF-α-, IL-17A-, or IL-22-induced pathway at the mRNA level.

## Discussion

The topical application of isoflavone extract prior to IMQ treatment significantly decreased the levels of TEWL and erythema, the speed of blood flow, and the thickness of the ear. The *in vitro* study demonstrated that, in human keratinocytes, cytokines such as TNF-α and IL-17A trigger the phosphorylation of the MAPK and NF-κB pathways and that IL-22 induces JAK/STAT and MAPK pathways, all of which induces inflammation. In the MAPK pathway, we found that the phosphorylation of signal transduction molecules such as ERK, p38 kinase, and JNK is upregulated by TNF-α, IL-17A, and IL-22. IκBα expression in the NF-κB pathway was also significantly upregulated after stimulation with TNF-α and IL-17A, and STAT3 and JAK2 expression in the JAK/STAT pathway was rapidly induced by IL-22. In contrast, pre-treatment with isoflavone extract downregulated the phosphorylation levels of p38 kinase, ERK, JNK, IκBα, STAT3, and JAK2.

Furthermore, the levels of CCL20 and the AMPs, includind S100A7, S100A8, S100A9, hBD2, and LL-37 alternate mRNA were analysed via qRT-PCR after treatment with the recombination proteins TNF-α, IL-17A, and IL-22 with or without pre-treatment with isoflavone extract. The results show that pre-treatment with isoflavone extract downregulated the levels of the AMPs.

The current study found that human Th17 pro-inflammatory cells produce IL-17A, IL-17F, TNF-α, IL-6, IL-21, IL-22, and IL-23^32^. Recent reports have discovered six isoform members of the IL-17 family, including IL-17A, IL-17B, IL-17C, IL-17D, IL-17E, and IL-17F. Several isoforms of the IL-17 receptor family have also been discovered, consisting of IL-17R, IL-17RH1, IL-17RL, IL-17RD, and IL-17RE^33^. Thus far, a number of autoimmune diseases are considered to be mediated by Th17, and the biological function of IL-17 is consistent with chronic inflammation and destructive inflammation responses. Other cell types are also involved in inflammation, including γδ T cells, natural killer T cells, natural killer cells, neutrophils, and eosinophils, all of which produce IL-17A and IL-17F. Both IL-17A and IL-17F are pro-inflammatory and interact with various cell types to induce the release of cytokines, such as transforming growth factor beta (TGF-β), tumour necrosis factor (TNF), interleukins (IL-1β, IL-6, IL-21, IL-23), granulocyte-macrophage colony-stimulating factor (GM-CSF), granulocyte colony-stimulating factor (G-CSF), chemokines (CXCL1, CXCL8, CXCL10), and metalloproteinase^34^. In addition, IL-17A and IL-17F are critical in neutrophils recruitment, activation, and migration^35^.

Interleukin-22 (IL-22) is a member of the IL-10 family, which consists of 179 residues and has 25% identity with human IL-10^36^. Human IL-22 is characterized by six alpha-helices that help maintain its secondary structure^37^. IL-22 was originally found to be stimulated by IL-19 in mice with T lymphocytes using the cDNA subtraction method, and is commonly considered an immunosuppressive cytokine, even though it can induce an immune response, as well. IL-22 has been shown to induce rheumatoid arthritis, along with an increase in IL-17 in patients’ plasma and synovial fluid, resulting in serious joint damage^38^. IL-22 also prevents inflammatory bowel disease (IBD) by producing a pancreatitis-associated protein in pancreatic acinar cells^39,40^, and can prevent autoimmune liver diseases, such as primary biliary cirrhosis (PBC). IL-22 also shows protective effects that ameliorate liver inflammation by decreasing T cell and B cell infiltration in PBC mice^41^. After IL-22 binds to the receptor complex on the cell membrane, which consists of two receptor chains, the IL-22R1 chain and the IL-10R2 chain^42^, it activates the JAK-STAT pathway^43,44^. Further, IL-22 regulates the expression of genes responsible for antimicrobial defence, cellular differentiation, mobility in keratinocytes^45^, and inhibition of epidermal differentiation^46^. These bioactivities suggest that IL-22 plays an important role in inflammatory skin processes and wound healing, but may be harmful to patients with psoriasis. The level of IL-22 was positively correlated with the production of IL-22 in plasma and the severity of psoriasis, indicating that IL-22 has a significant effect on psoriasis^47^. Besides that, high IL-22 levels in psoriatic skin were associated with strongly upregulated cutaneous S100A7, S100A8, S100A9, and MMP1 expression^45^.

TNF-α is the primary mediator of inflammatory skin disease pathology. It is a multifunctional cytokine that plays an important role in inflammation, immune response, and apoptosis^48^. Patients with psoriasis have higher levels of TNF-α and translation factors such as NF-κB in their skin lesions^49,50^. This is due to TNF-α’s ability to trigger NF-κB activation, which leads to the activation of other cytokines and upregulation of TNF-α itself, in a positive feedback loop^49,51^. In addition, TNF-α causes mTOR activation and ROS generation, which leads to IκB degradation, NF-κB translocation, and, finally, the production and secretion of inflammatory cytokines^52^. Moreover, TNF-α was shown to activate the Ras/Raf/MEK/ERK and protein kinase B/Akt pathways. In a human epidermal cell line, phosphatidylinositol (PI)3-kinase/Akt transduction was activated by TNF-α, which subsequently triggered the activation of NF-κB, which, in turn, regulated gene expression, and induced innate and adaptive immune responses and inflammation^53^. TNF-α also induces phosphorylation of JNK and reduces the expression of filaggrin and loricrin–two major proteins expressed by terminally differentiated epidermal keratinocytes^54^. Therefore, the use of a TNF-α antagonist may increase the expression of the skin barrier and ameliorate the symptoms of psoriasis by improving the barrier protein expression^55^. Antioxidants, also, have been shown to reduce the TNF-α-induced production of cytokines^52^, and may, therefore, play an important role in inflammatory skin diseases, especially psoriasis^52^. It is reasonable to assume that antioxidants have the potential to be developed into a new drug which would have both anti-inflammatory and barrier repair properties.

Skin is the body’s largest organ^56^ and serves as the main barrier to the body’s internal and external environment in order to maintain body homeostasis^57^, and to protect the human body from external damage. In addition, the skin can recognize, discriminate, and integrate various signals from the outside environment^58^, choose appropriate responses, and trigger a variety of physiological reactions including skin immune, pigmentary, epidermal, adnexal systems, systemic immune, neural, and endocrine systems^59–62^. In previous studies, it was reported that many skin-related responses are mediated via the cutaneous neuroendocrine system^63^. For example, in response to environmental changes, the skin can generate signals to produce neural, humoral, or immune responses at the local and systemic levels. In this study, we explored the effect of inflammatory-related molecules on the mechanisms of psoriasis. In the future, we hope to study the cutaneous neuroendocrine system in more detail.

Flavonoids have been widely investigated in the past. One flavonoid, epigallocatechin gallate (EGCG), has been reported to possess anti-inflammatory effects in the skin. EGCG has been found effective against UVB-induced prostaglandin metabolism, and also to reduce UVB-induced erythema, myeloperoxidase activity, hydrogen peroxide generation, and leukocyte infiltration^64,65^. In addition, EGCG inhibits UVB-induced AP-1 upregulation and the expression of apoptosis-regulatory genes (p53-p21)^66^, and possesses a protective effect on UVA-induced damage in human keratinocytes^67^. Similar to EGCG, (−)-epicatechin-3-gallate (ECG) can protect against UVA-induced cell death of human keratinocytes by reducing the generation of H_2_O_2_ and hypoxanthine-xanthine oxidase^68,69^. Our previous research also found that (+)-catechin provides an anti-oxidation effect after UVB exposure, but the mechanism of (+)-catechin differs from that of EGCG, since (+)-catechin protects against UV and hydrogen peroxide damage via inhibition of JNK phosphorylation^70^. Genistein, found in soybeans, can prevent UVB-induced H_2_O_2_ generation, lipid peroxidation, and oxidative DNA damage, and inhibits the progression and promotion of cancer via the inhibition of tyrosine protein kinase^71^. Further studies have shown that genistein can also suppress the expression of UVB-induced *c-fos* and *c-jun* proto-oncogenes, inhibiting the transformation of a normal cell into a tumour cell^72^.

Previous studies in our laboratory have shown that isoflavone extract works better against photo-aging (UVB) than genistein and is almost non-toxic. These two properties give isoflavones excellent potential for therapeutic applications and development in the future^73,74^. Theaflavins, the main component of black tea, are also reported to possess excellent antioxidant activity along with the ability to downregulate UV-induced lipid oxidation to reduce skin damage further^75^. Theaflavins also suppress the activation of transcription factor AP-1^76^, UVB-induced phosphorylation of STAT1 at Ser727^77^, and arsenite-induced apoptosis^78^. In another study, 12-o-tetradecanoylphorbol-13-acetate (TPA)-induced inflammation in a rodent model showed that theaflavins reduced ornithine decarboxylase (ODC) levels, which led to an anti-inflammatory effect^79^, and decreased NF-κB phosphorylation, thereby inhibiting tumour production^76^.

Another plant, indigo naturalis has been used as a medicine for the treatment of skin diseases in the past. In recent years, more and more reports have shown that indigo naturalis and indirubin can be effectively and safely used for the treatment of psoriasis^80^, especially nail psoriasis and plaque-type psoriasis^81–83^. Indigo naturalis upregulates claudin-1 expression and tight-junctions in human keratinocytes and also has a strong inhibitory effect on the inflammatory response of human neutrophils^84,85^. Therefore, an exploration of the relationship between natural products and activity will be of great value to the development of anti-inflammatory and barrier repair skin treatments.

The use of steroids can suppress both the immune system and inflammation, but it does not cure the source of the inflammation. When the use of steroids is discontinued, the inflammatory response will flare up again, unless the allergens causing the skin inflammation have been neutralized. Moreover, long-term and significant steroid use inevitably leads to systemic side effects, since steroids absorbed by the body inhibit the normal secretion of epinephrine. The long-term result is a reduction in pituitary and adrenal gland secretions, which causes dermis layer thinning that does not return to normal even after cessation of steroid use, microvascular proliferation, loss of melanin, and skin that begins to fade^86^. Thus, the use of steroids involves many limitations, while the use of natural products and Chinese herbal medicines is possible at a low cost, without significant restrictions, or side effects, which is a significant advantage in the treatment of inflammatory skin diseases such as psoriasis.

## Materials and Methods

### Ethics Statement

The foreskins used in the experiments was provided by the Mackay Memorial Hospital to the Fu Jen Catholic University with a letter providing an exemption from the institutional review board (IRB) (#13MMHIS022), as no interaction occurred with the foreskin donors themselves and no identifiable information was made available to the researchers. All animal tests were approved by the Fu Jen Catholic University’s Institutional Animal Care and Use Committee (IACUC) policy (approval #A10367).

### Animals

Mice were obtained from the National Laboratory Animal Center, Taipei, Taiwan. Animals were housed and handled according to institutional guidelines. Briefly, mice were housed one per cage in a controlled environment for 1 week with the temperature set at 21–25 °C, humidity at 60 ± 5%, and light in a 12/12 h light/dark cycle. Alfalfa-free food and water were given ad libitum. All of the animal-experiment protocols were reviewed by the committee and conducted after obtaining an Affidavit of Approval of Animal Use Protocol from Fu Jen Catholic University.

### IMQ-induced psoriasis-like skin inflammation in mice

Male BALB/c mice (8–11 weeks) received a daily topical dose of 62.5 mg of commercially available IMQ cream (Aldara 5%; Meda AB, Solna, Sweden) on shaved areas of the dorsal and lumbar back and on the right ear for 6 consecutive days. Similarly, a vehicle cream (Vaselina Pura, Laboratorios Rida, Valencia, Spain) was applied to the IMQ-untreated mice. The trans-epidermal water loss (TEWL), amount of erythema, amount of melanin, skin hydration, pH value, ear thickness, and blood flow were measured before treatment. The surface changes in the dorsal skin were recorded using photography. The TEWL, erythema, melanin level, skin hydration, and blood flow were regularly measured using an MPA-580 cutometer (Courage & Khazaka, Cologne, Germany) and FLO-N1 laser tissue blood flowmeter (Omegawave, Tokyo, Japan). One hour before the cream was applied, isoflavone extract (10 mg/mL) or vehicle were topically administered. At the end of the experiment, the mice were killed, and tissues were collected and stored at −80 °C for subsequent homogenization and fixture in formalin. Animals were removed from the study and euthanized with CO_2_ when clear suffering negated the need to continue, in accordance with the Fu Jen Catholic University’s IACUC policy.

### Histopathological analysis

Mouse tissues were fixed overnight with neutral buffered 4% paraformaldehyde (PFA) at 4 °C and then directly embedded in paraffin. Blocks of skin biopsies in paraffin were prepared using routine methods and sectioned to obtain consecutive levels. Five-micrometre sections were stained either with haematoxylin and eosin (H&E) or processed further. Images from H&E staining were obtained using a ZEISS Axioskop 40 Inverted System Microscope (NY, United States) and SPOT Cam software (Sterling Heights, MI).

### Primary human keratinocyte isolation from foreskin and cell culture

Normal human epidermal keratinocytes (NHEKs) were obtained from normal adult human foreskins. All protocols and procedures were approved by the local ethics committee and carried out in accordance with the Declaration of Helsinki. Written consent was obtained from each donor before the experiments were performed. In brief, the skin was divided and incubated in 0.2% protease (Sigma-Aldrich, St Louis, MO) for 2 days at 4 °C. After the epidermis separated from the dermis, the keratinocytes were disaggregated into a single-cell suspension in serum-free Dulbecco’s modified Eagle’s medium (DMEM) and then centrifuged at 1100 × rpm for 5 minutes. The keratinocytes were then cultured in Keratinocyte-SFM (Gibco BRL/Invitrogen, Carlsbad, CA), supplemented with recombinant epidermal growth factor (0.1–0.2 ng·mL^−1^), bovine pituitary extract (20–30 μg·mL^−1^), and 1% penicillin/streptomycin in a humidified atmosphere at 37 °C and 5% CO_2_. The second- to fourth-passage cells were used in the experiments.

### Stimulation of primary human keratinocytes

NHEKs were seeded in 12-well plates at a density of 5 × 10^4^ cells/well. After reaching subconfluency, the keratinocyte medium was renewed, and the cells were subjected to a 24 h-pretreatment with isoflavone extract. Control keratinocytes were placed in an equal volume of vehicle. Finally, cells were stimulated with either IL-17A (50 ng·mL^−1^), IL-22 (50 ng·mL^−1^), or TNF-α (50 ng·mL^−1^) from PeproTech (Rocky Hill, NJ).

### Cell viability assays (MTT, trypan blue assay, and crystal violet assay)

Cell viability was determined via MTT, trypan blue, and crystal violet assays. The MTT assay was performed according to a previously described protocol^68^. Briefly, cells were pretreated with double-distilled (dd)H_2_O or isoflavone extract and incubated for 24 hours. After being washed with the phosphate buffer solution (PBS), MTT (0.5 mg/mL in Keratinocyte-SFM) was used for the quantification of metabolically active cells. Mitochondrial dehydrogenases metabolized MTT to a purple formazan dye, which was analysed photometrically at 550 nm. Cell viability was proportional to the absorbance.

The Trypan Blue exclusion method was used, according to the manufacturer’s protocols, to accurately determine cell viability subsequent to isolation. Briefly, NHEKs were seeded in a 35 mm culture dish. After reaching subconfluency, the Keratinocyte-SFM was renewed, and the cells were subjected to a 24-h pretreatment with isoflavone extract. The cells were then collected into cellular suspension with TrypLE Express (Gibco BRL/Invitrogen, Carlsbad, CA) and stained with equal volumes of 0.4% trypan blue dye for 1 min. The cells were counted using a dual-chamber haemocytometer and a light microscope.

The crystal violet assay was used to determining the viability of cultured cells^87^. Crystal violet is a triarylmethane dye (purple) that stains DNA and proteins in cells that are grown into a monolayer. The cytotoxicity of a drug is determined by measuring the absorbance of the crystal violet-stained cells via spectrophotometry. Cell treatments were performed as above described. After a 24-h incubation period, the cells were washed with PBS to remove non-adherent cells. Then, methanol (500 μL) was added to each well to fix the living cells to the bottom of the plate, and the cells were incubated for 30 min at 25 °C. The methanol was discarded and 0.1% crystal violet staining solution (300 μL) was added for 1 h. The crystal violet was discarded, and the plate was washed three times with ddH_2_O. The plates were left to dry and 33% acetic acid (500 μL) was added to dissolve the stained cells. Absorbance optical density (OD) was read using a Tecan Sunrise spectrophotometer (Tecan, Crailsheim, Germany) at a wavelength of 550 nm.

### Cell lysate preparation and western blot analysis

NHEKs treated with or without cytokines were washed twice with PBS and lysed with radioimmunoprecipitation assay (RIPA) buffer [17 mM Tris–HCl, pH 7.4, 50 mM NaCl, 5 mM EDTA, 1 mM sodium fluoride, 1% Triton X-100, 1% sodium deoxycholate, 0.1% sodium dodecyl sulphate (SDS), 1 mM sodium orthovanadate, 1 mM phenylmethane sulfonyl fluoride (PMSF), 1 μg/mL aprotinin (freshly prepared), and 1 μg/mL leupeptin (freshly prepared)]. After sonication, the lysate was centrifuged (14,000 × *g* for 10 min at 4 °C), and the supernatant was removed. The protein content was quantified using a Pierce protein assay kit (Pierce, Rockford, IL). The total protein was separated via electrophoresis on 10% SDS–polyacrylamide gels. The proteins were then electroblotted onto polyvinylidene fluoride (PVDF) membranes and probed using the indicated specific antibodies. Immunoblots were detected via enhanced chemiluminescence (Chemiluminescence Reagent Plus, NEN, Boston, MA). For some of the experiments, the PVDF membrane was stripped at 60°C for 10 minutes with a stripping buffer (62.5 mM Tris-HCl, pH 6.7, 2% SDS, and 100 mM β-mercaptoethanol).

### Real-time quantitative reverse transcriptase – polymerase chain reaction (RT-qPCR)

After these treatments, the cells were lysed and the total RNA was extracted using a total RNA isolation kit (GeneDireX^®^, Vegas, NV). First strand cDNAs were synthesized using the SuperScript^®^ III First-Strand Synthesis System (Invitrogen, Carlsbad, CA), according to the manufacturer’s instructions. qPCR was performed using an CFX96™ Real-Time PCR Detection System (Bio-Rad, Hercules, CA) and under the following conditions: heat to 95 °C for 3 min, followed by 54 denaturation cycles at 95 °C for 3 s each, primer annealing at 60 °C for 20 s, and primer extension at 95 °C for 10 s. The melting temperature curve ranged from 60 to 95 °C, increasing in increments of 0.3 °C. cDNA was amplified using SYBR green (Kapa Biosystems, Wilmington, MA) and using the following primers: human CCL20^88^, forward TACTCCACCTCTGCGGCGAATCAGAA, and reverse GTGAAACCTCCAACCCCAGCAAGGTT; human S100A7^89^, forward GCATGATCGACATGTTTCACAAATACAC, and reverse TGGTAGTCTGTGGCTATGTCTCCC; human S100A8^90^, forward TGAAGAAATTGCTAGAGAC, and reverse CTTTATCACCAGAATGAGGA; human S100A9^46^, forward GCTCCTCGGCTTTGACAGAGTGCAAG, and reverse GCATTTGTGTCCAGGTCCTCCATGATGTGT; human BD2^91^, forward CCAGCCATCAGCCATGAGGGT, and reverse GGAGCCCTTTCTGAATCCGCA; human LL-37^92^, forward GCAGTCACCAGAGGATTGTGAC, and reverse CACCGCTTCACCAGCCC; human β-Actin^93,94^, forward CGGGGACCTGACTGACTACC, and reverse AGGAAGGCTGGAAGAGTGC. The CCL20, S100A7 to 9, hBD2, and LL-37 amplification signals were normalized relative to β-actin expression and evaluated using the equation: fold change = 2^−ΔΔCT^.

### Statistical analysis

Unless otherwise indicated, data are expressed as mean ± standard error of the mean (SEM) using the GraphPad Prism Program 6 software (GraphPad Software, San Diego, CA). Comparison of the mean survival rates of cells with and without isoflavone extract was performed using a one-way analysis of variance (ANOVA) followed by Dunnett’s t-test for multiple comparisons. We considered P < 0.05 to be statistically significant.
